# Bounds for coherence of quantum superpositions in high dimension

**DOI:** 10.1038/s41598-017-03885-5

**Published:** 2017-06-21

**Authors:** Qiu-Ling Yue, Fei Gao, Qiao-Yan Wen, Wei-Wei Zhang

**Affiliations:** 1 State Key Laboratory of Networking and Switching Technology, Beijing University of Posts and Telecommunications, Beijing, 100876 China; 2 State Key Laboratory of Information Security, (Institute of Information Engineering, Chinese Academy of Sciences), Beijing, 100093 China; 3 Hefei National Laboratory for Physical Sciences at Microscale, University of Science and Technology of China, Hefei, Anhui 230026 China

## Abstract

Quantum coherence plays a major role in the promotion for quantum information processing and designing quantum technology. Since coherence is rooted in superposition principle, it is vital to understand the coherence change with respect to superpositions. Here we study the bounds for coherence of quantum superpositions in high dimension. We consider three most frequently used measures of coherence, i.e. the relative entropy of coherence, *l*_1_ norm of coherence and robustness of coherence. For a quantum state (an arbitrary dimension) and its arbitrary decomposition, we give the upper and lower bounds for coherence of the superposition state in terms of the coherence of the states being superposed.

## Introduction

Quantum coherence is a fundamental feature of quantum mechanics. As one of the most crucial physical resources, it plays a primary role in quantum information processing^1–3^, computational task^4, 5^, quantum metrology^6, 7^, thermodynamics^8–10^, and quantum biology^11, 12^. The theory of coherence as a resource was first set forth in ref. 13, where the authors introduced a rigorous framework for the quantification of coherence and identified the computable measures of coherence. From resource-driven viewpoint, there is a growing number of work studying coherence including different coherence measures^14–18^, the properties of coherence^19, 20^, the freezing phenomenon of coherence^21, 22^, the relation among coherence, entanglement and quantum correlation^23–25^, and so on refs 26, 27.

Despite coherence derives from the superposition of states, the coherence of a superposition state cannot be directly deduced from the coherence of the individual states being superposed. We illustrate it with the following examples: given a state

1 $$|{{\rm{\Omega }}}_{1}\rangle =\frac{{\rm{1}}}{\sqrt{{\rm{2}}}}(|0\rangle +|1\rangle ),$$

it is obvious that the coherence of |0〉, |1〉 is 0, while the coherence of |Ω_1_〉 reaches maximum value. Here, we consider coherence in the computational basis. In the following, we show an opposite example. Given a state

2 $$|{{\rm{\Omega }}}_{2}\rangle =\frac{1}{\sqrt{2}}(|+\rangle +|-\rangle ),$$

where $|\pm \rangle =(|0\rangle \pm |1\rangle )/\sqrt{2}$. Interestingly, under computational basis the coherence of |+〉, |−〉 reaches maximum value while the coherence of their superposition |Ω_2_〉 is 0.

Our concern here is that: given an arbitrary state |Ω〉 and its arbitrary decomposition

3 $$|{\rm{\Omega }}\rangle =\alpha |{\rm{\Phi }}\rangle +\beta |{\rm{\Psi }}\rangle ,$$

what is the relation between the coherence of |Ω〉 and the coherence of |Φ〉 and |Ψ〉? Because of the importance of coherence in quantum physics and superposition for coherence, the solutions for this problem will provide a theoretical foundation for potential applications of quantum resource and quantum information processing.

Similar problem has been studied in the field of entanglement. In 2006, Linden *et al*. firstly studied the relation between the entanglement of |Ω〉 and the entanglement of |Φ〉 and |Ψ〉^28^, in which they gave the upper bounds on the entanglement of the superposition state in terms of the entanglement of the states being superposed using von Neumann entropy of the reduced state as a measure of entanglement. Thereafter, there are some related works. Ref. 29 gave a tighter upper bound on the same question and also gave a lower bound. Refs 30, 31 studied the same problem considering concurrence (another measure of entanglement) and gave the corresponding upper and lower bound. However, this problem is still open in the field of coherence where only two specific cases have been discussed^32, 33^.

In this work, we analyze the relation between the coherence of |Ω〉 and the coherence of its decomposition. We systematically study this problem considering three kinds of coherence measure, and give the corresponding tight upper and lower bounds. Our results can be used for estimating the coherence range of the superposition state. For example, given the coherence of two states, we do not even need to know what the state is, we can estimate the range of the coherence resource we can get from their superposition state. In addition, armed with these relationships on coherence of superpositions, we can easily monitor the coherence change in the quantum information processing, such as coherence distillation. Coherence is likewise a measure of information carrying ability. The more coherence, the more information can be carried in the states.

In our work, we focus on orthogonal version of problem (i.e. |Φ〉 and |Ψ〉 are orthogonal states). We can get the non-orthogonal version of the problem easily by the following decomposition,

4 $$|{\rm{\Omega }}\rangle =\alpha |{\rm{\Phi }}\rangle +\beta |{\rm{\Psi }}\rangle =\alpha |{\rm{\Phi }}\rangle +\beta (\gamma |{\rm{\Phi }}\rangle +\sqrt{1-\gamma }|{{\rm{\Phi }}}^{\perp }\rangle ),$$

where |Φ〉 and |Φ^⊥^〉 are orthogonal states.

## Results

### Relative entropy of coherence

A well-defined and frequently used coherence measure is the relative entropy of coherence, which is proposed and studied in ref. 13. With a particular entropic formula, the relative entropy of coherence has some clear physical meanings, such as it is equal to the optimal distillation rate for standard coherence distillation^34^, and can also be interpreted as the minimal amount of noise required for fully decohering the state^27, 35^. In this section, we study the relationship between the coherence of two orthogonal states and the coherence of its decomposition using the relative entropy of coherence.

Given a particular basis $\{{|i\rangle }_{i=1}^{n}\}$, the definition of relative entropy of coherence^13^ is

5 $${C}_{re}(\rho )=S({\rho }_{d})-S(\rho ),$$

where *ρ* is density operator and *ρ*_*d*_ denotes the state obtained from *ρ* by deleting all off-diagonal elements under the particular basis, and *S*(*ρ*) is von Neumann entropy of *ρ*. In the case of a pure state |*ϕ*〉, its relative entropy of coherence can be expressed as

6 $${C}_{re}(\varphi )=S({|\varphi \rangle }_{d}\langle \varphi |).$$

In the following, we will give the bounds with respect to relative entropy of coherence. The proof of this Theorem is in Methods.

**Theorem 1**. *Given two orthogonal states* |Φ〉, |Ψ〉, *and two complex number α*, *β satisfying* $\Vert \alpha |{\rm{\Phi }}\rangle +\beta |{\rm{\Psi }}\rangle \Vert =1$, *the coherence of the superposition* $|{\rm{\Omega }}\rangle =\alpha |{\rm{\Phi }}\rangle +\beta |{\rm{\Psi }}\rangle$ *satisfies*

7 $${C}_{re}({\rm{\Omega }})\le \,{\rm{\min }}\{\begin{array}{l}\tfrac{1}{q}f(p),\\ f(p)+2(1-q)|\alpha ||\beta |\,\mathrm{log}(n-1)+(1-q),\end{array}$$

*for* 0 < *p* < 1, *where*

$$q=\frac{p\mathrm{(1}-p)}{\mathrm{(1}-p){|\alpha |}^{2}+p{|\beta |}^{2}},\,f(p)=p{C}_{re}({\rm{\Phi }})+(1-p){C}_{re}({\rm{\Psi }})+{h}_{2}(p),$$

${h}_{2}(x)\equiv -x\,\mathrm{log}\,x-(1-x)\,\mathrm{log}\,\mathrm{(1}-x)$, *and n is the dimension of Hilbert space*.

*Remark* 1: The result in ref. 32 is the case with *p* = |*α*|^2^ in Theorem 1.

*Remark* 2: We give an example to show the upper bound in Theorem 1 is tight in some cases.

*Example* 1: Consider the following case:

8 $$\begin{array}{rcl}|{\rm{\Phi }}\rangle  & = & \frac{1}{\sqrt{2}}(|1\rangle +\frac{1}{\sqrt{n-1}}(|2\rangle +|3\rangle +\cdots +|n\rangle )),\\ |{\rm{\Psi }}\rangle  & = & \frac{1}{\sqrt{2}}(|1\rangle -\frac{1}{\sqrt{n-1}}(|2\rangle +|3\rangle +\cdots +|n\rangle )),\\ \,\,\alpha  & = & -\beta =\frac{1}{\sqrt{2}},\end{array}$$

where *n* is the dimension of Hilbert space.

In this case, we can see that the upper bound in Theorem 1 is tight. Considering *p* = |*α*|^2^, the first bound in Eq. (7) equals to $2[{|\alpha |}^{2}{C}_{re}({\rm{\Phi }})+{|\beta |}^{2}{C}_{re}({\rm{\Psi }})+{h}_{2}({|\alpha |}^{2})]$. The coherence of |Φ〉 and |Ψ〉 both equal to $\frac{1}{2}\,\mathrm{log}\,(n-\mathrm{1)}+1$, and the coherence of their superposition state $|{\rm{\Omega }}\rangle =\alpha |{\rm{\Phi }}\rangle +\beta |{\rm{\Psi }}\rangle$ is log (*n* − 1). The ratio of *C*_*re*_(Ω) and ${|\alpha |}^{2}{C}_{re}({\rm{\Phi }})+{|\beta |}^{2}{C}_{re}({\rm{\Psi }})+{h}_{2}({|\alpha |}^{2})$ converges to 2 when *n* is infinite as shown in Eq. (9).

9 $$\mathop{\mathrm{lim}}\limits_{n\to \infty }\frac{{C}_{re}({\rm{\Omega }})}{2[{|\alpha |}^{2}{C}_{re}({\rm{\Phi }})+{|\beta |}^{2}{C}_{re}({\rm{\Psi }})+{h}_{2}({|\alpha |}^{2})]}=1.$$

**Figure 1:**
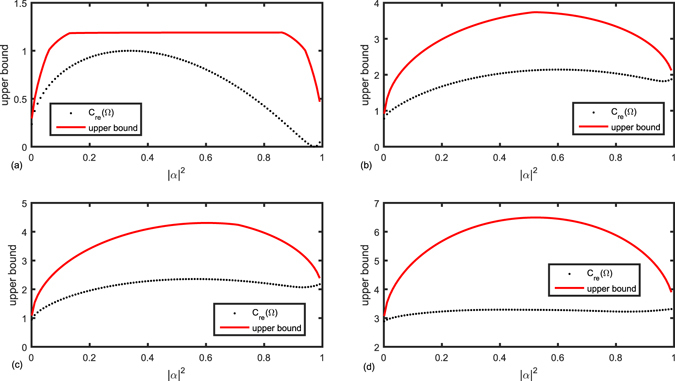
The upper bounds on coherence of the superpositions for dimension = 2, 5, 7, 15, with |Φ〉 and |Ψ〉 as defined in the text. The black dotted line is the exact value of *C*_*re*_(Ω); the red solid one is the upper bound in Theorem 1. Note that both *α* and *β* are chosen to be positive numbers here.

We give numerical simulations for the upper bound in Theorem 1 and the exact coherence of |Ω〉 in different dimensions by choosing two random orthogonal states |Φ〉 and |Ψ〉 as shown in Fig. 1. The sub-figures (a–d) represent the comparison in different dimensions (2, 5, 7, 15 respectively). Let |Φ〉 and |Ψ〉 are chosen randomly as following: (a) $|{\rm{\Phi }}\rangle =0.9863|0\rangle -0.1650|1\rangle$, and $|{\rm{\Psi }}\rangle =0.1650|0\rangle +0.9863|1\rangle$; (b) $|{\rm{\Phi }}\rangle =0.6252|0\rangle -0.4357|1\rangle$ $-0.1846|2\rangle -0.5545|3\rangle +0.2788|4\rangle$, and $|{\rm{\Psi }}\rangle =0.2079|0\rangle +0.1082|1\rangle$ $+0.9322|2\rangle -0.0226|3\rangle +0.2750|4\rangle$; (c) $|{\rm{\Phi }}\rangle =-0.2503|0\rangle +0.1711|1\rangle +0.2273|2\rangle +0.6442|3\rangle$ + $0.2732|4\rangle +0.5262|5\rangle +0.2998|6\rangle$,and $|{\rm{\Psi }}\rangle =0.9139|0\rangle +0.0736|1\rangle +0.2927|2\rangle$ + $0.0612|3\rangle -0.0641|4\rangle +0.2555|5\rangle -0.0224|6\rangle$; (d) $|{\rm{\Phi }}\rangle =0.3266|0\rangle$ $+0.1666|1\rangle -0.1765|2\rangle +0.2515|3\rangle$ + $0.4549|4\rangle +0.1634|5\rangle -0.2075|6\rangle -0.2327|7\rangle +0.0535|8\rangle$  − $0.1524|9\rangle$ $-0.4818|10\rangle +0.1975|11\rangle$ + $0.0855|12\rangle -0.2922|13\rangle -0.2244|14\rangle$, and$|{\rm{\Psi }}\rangle =0.1829|0\rangle +0.4304|1\rangle +0.0664|2\rangle +$ $0.0460|3\rangle +0.1304|4\rangle$ + $0.3446|5\rangle +0.0927|6\rangle +0.0213|7\rangle +0.1006|8\rangle +0.4598|9\rangle$ + $0.0060|10\rangle +0.0322|11\rangle$ $+0.3234|12\rangle +0.2864|13\rangle +0.4698|14\rangle$.

The black dotted line is the exact coherence of superposition state |Ω〉, the red solid one is the upper bound in Theorem 1. Note that *α* and *β* we chose are both positive numbers. From this figure, we can see that given the state |Φ〉 and |Ψ〉, the upper bound in Theorem 1 depends on the parameter *α*. Parallelly, given the value of *α*, the coherence of states |Φ〉 and |Ψ〉 also affects upper bound in Theorem 1.

Now, we move to analyze the lower bounds. By constructing a special state and measuring it with an incoherent operation, we can get the lower bound for coherence of superpositions considering relative entropy of coherence as stated in following theorem. Its detailed proof is in Methods.

**Theorem 2**. *Given two orthogonal states* |Φ〉, |Ψ〉, *and two complex number α*, *β satisfying* $\Vert \alpha |{\rm{\Phi }}\rangle +\beta |{\rm{\Psi }}\rangle \Vert =1$, *the coherence of the superposition* $|{\rm{\Omega }}\rangle =\alpha |{\rm{\Phi }}\rangle +\beta |{\rm{\Psi }}\rangle$ *satisfies*

10 $$\begin{array}{rcl}{C}_{re}({\rm{\Omega }}) & \ge  & {\rm{\max }}\{t{C}_{re}({\rm{\Phi }})-\frac{1-p}{p}{C}_{re}({\rm{\Psi }})\\  &  & -\frac{1}{p}{h}_{2}(p),t\in \{\frac{\mathrm{(1}-p){|\alpha |}^{2}}{1-p{|\alpha |}^{2}},\frac{\mathrm{(1}-p){|\beta |}^{2}}{1-p{|\beta |}^{2}}\}\},\end{array}$$

*for* 0 < *p* < 1, *and* $${h}_{2}(x)\equiv -x\,\mathrm{log}\,x-\mathrm{(1}-x)\,\mathrm{log}\,\mathrm{(1}-x)$$.

### *l*_1_ norm coherence

Another measure of coherence, *l*_1_ norm, is defined with the off-diagonal elements of the considered quantum state. This definition is intuitive for the measure of coherence and satisfies the necessary properties presented in ref. 13.

Given a fixed basis $\{{|i\rangle }_{i=1}^{n}\}$, where *n* is the dimension of Hilbert space. The definition of *l*_1_ norm of coherence of a state *ρ* is ref. 13

11 $${C}_{{l}_{1}}(\rho )\equiv \sum _{i\ne j}\,|{\rho }_{ij}|.$$

Specially, for a pure state $|\varphi \rangle =\sum \,{a}_{i}|i\rangle$, the *l*_1_ norm of coherence is

12 $${C}_{{l}_{1}}(\varphi )\equiv \sum _{i\ne j}\,|{a}_{i}{a}_{j}|.$$

It can also be expressed as

13 $${C}_{{l}_{1}}(\varphi )\equiv {(\sum _{i}|{a}_{i}|)}^{2}-1.$$

We consider *l*_1_ norm of coherence measure and obtain corresponding bounds for coherence of superpositions. See Methods for the proof of the following Theorem.

**Theorem 3**. *Given two orthogonal states* |Φ〉, |Ψ〉, *and two complex number α*, *β satisfying* $\Vert \alpha |{\rm{\Phi }}\rangle +\beta |{\rm{\Psi }}\rangle \Vert =1$, *the coherence of the superposition* $|{\rm{\Omega }}\rangle =\alpha |{\rm{\Phi }}\rangle +\beta |{\rm{\Psi }}\rangle$ *satisfies*

14 $$\begin{array}{c}{C}_{{l}_{1}}({\rm{\Omega }})\le \,min\{\begin{array}{c}{|\alpha |}^{2}{C}_{{l}_{1}}({\rm{\Phi }})+{|\beta |}^{2}{C}_{{l}_{1}}({\rm{\Psi }})+2|\alpha \beta |(n-1),\\ {|\alpha |}^{2}{C}_{{l}_{1}}({\rm{\Phi }})+{|\beta |}^{2}{C}_{{l}_{1}}({\rm{\Psi }})+2|\alpha \beta |\sqrt{({C}_{{l}_{1}}({\rm{\Phi }})+1)({C}_{{l}_{1}}({\rm{\Psi }})+1)},\end{array}\end{array}$$

*where n is the dimension of Hilbert space*.

*Remark* 3: An interesting symmetric inequality can be deduced from the second line in Eq. (14) as follows:

15 $$\sqrt{{C}_{{l}_{1}}({\rm{\Omega }})+1}\le |\alpha |\sqrt{{C}_{{l}_{1}}({\rm{\Phi }})+1}+|\beta |\sqrt{{C}_{{l}_{1}}({\rm{\Psi }})+1}.$$

This inequality may have applications in other analysis of coherence by considering superposition effects.

*Remark* 4: Here, we want to emphasize that in Theorem 3 the dimension *n* is an arbitrary positive integer and our result covers the two dimension case mentioned in ref. 33.

**Figure 2:**
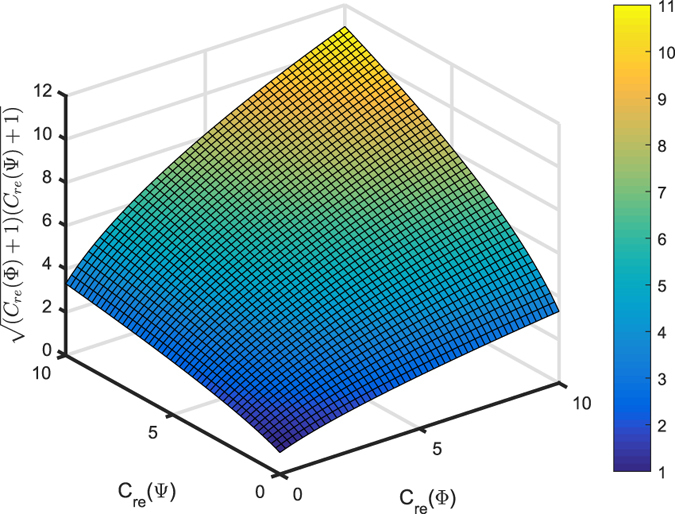
The comparison between $\sqrt{({C}_{{l}_{1}}({\rm{\Phi }})+1)({C}_{{l}_{1}}({\rm{\Psi }})+1)}$ and *n* − 1. Here the domains of ${C}_{{l}_{1}}({\rm{\Phi }})$ and ${C}_{{l}_{1}}({\rm{\Psi }})$ are set as[0, 10].

Now, we compare the values of two expressions in Theorem 3. The only difference between them locates at the last term. One is 2|*αβ*| (*n* − 1), and the other is $2|\alpha \beta |\sqrt{({C}_{{l}_{1}}({\rm{\Phi }})+1)({C}_{{l}_{1}}({\rm{\Psi }})+1)}$. Thus, we only need to compare the values of *n* − 1 and $\sqrt{({C}_{{l}_{1}}({\rm{\Phi }})+1)({C}_{{l}_{1}}({\rm{\Psi }})+1)}$. In Figure 2, we plot function $\sqrt{({C}_{{l}_{1}}({\rm{\Phi }})+1)({C}_{{l}_{1}}({\rm{\Psi }})+1)}$ by setting ${C}_{{l}_{1}}({\rm{\Phi }})$ and ${C}_{{l}_{1}}({\rm{\Psi }})$ as independent variables whose domains are[0, 10]. Since *n* − 1 is an integer, it is easy for us to compare the value of $\sqrt{({C}_{{l}_{1}}({\rm{\Phi }})+1)({C}_{{l}_{1}}({\rm{\Psi }})+1)}$ and coordinate axis scale which can be recognized as *n* − 1. If *n* − 1 is smaller than the value of $\sqrt{({C}_{{l}_{1}}({\rm{\Phi }})+1)({C}_{{l}_{1}}({\rm{\Psi }})+1)}$, then the upper bound will be ${|\alpha |}^{2}{C}_{{l}_{1}}({\rm{\Phi }})+{|\beta |}^{2}{C}_{{l}_{1}}({\rm{\Psi }})+2|\alpha \beta |(n-\mathrm{1)}$. If *n* − 1 is larger than the value of $\sqrt{({C}_{{l}_{1}}({\rm{\Phi }})+1)({C}_{{l}_{1}}({\rm{\Psi }})+1)}$, the ${|\alpha |}^{2}{C}_{{l}_{1}}({\rm{\Phi }})+{|\beta |}^{2}{C}_{{l}_{1}}({\rm{\Psi }})+2|\alpha \beta |\sqrt{({C}_{{l}_{1}}({\rm{\Phi }})+1)({C}_{{l}_{1}}({\rm{\Psi }})+1)}$ is the tighter upper bound.

Next, we focus on discussing lower bound. Through using the absolute value inequality, we can obtain the lower bound for *l*_1_ norm of coherence of superpositions as illustrated in the following theorem. Its proof can be found in Methods.

**Theorem 4**. *Given two orthogonal states* |Φ〉, |Ψ〉, *and two complex number α*, *β satisfying* $\Vert \alpha |{\rm{\Phi }}\rangle +\beta |{\rm{\Psi }}\rangle \Vert =1$, *the coherence of the superposition* $|{\rm{\Omega }}\rangle =\alpha |{\rm{\Phi }}\rangle +\beta |{\rm{\Psi }}\rangle$ *satisfies*

16 $$\begin{array}{c}{C}_{{l}_{1}}({\rm{\Omega }})\ge \,{\rm{\max }}\{\begin{array}{l}{|\alpha |}^{2}{C}_{{l}_{1}}({\rm{\Phi }})+{|\beta |}^{2}{C}_{{l}_{1}}({\rm{\Psi }})-2|\alpha \beta |(n-1),\\ {|\alpha |}^{2}{C}_{{l}_{1}}({\rm{\Phi }})+{|\beta |}^{2}{C}_{{l}_{1}}({\rm{\Psi }})-2|\alpha \beta |\sqrt{({C}_{{l}_{1}}({\rm{\Phi }})+1)({C}_{{l}_{1}}({\rm{\Psi }})+1)},\\ 0,\end{array}\end{array}$$

*where n is the dimension of Hilbert space*.

*Remark* 5: An interesting symmetric inequality can be deduced from the second line in Eq. (16) as follows:

17 $$\sqrt{{C}_{{l}_{1}}({\rm{\Omega }})+1}\ge ||\alpha |\sqrt{{C}_{{l}_{1}}({\rm{\Phi }})+1}-|\beta |\sqrt{{C}_{{l}_{1}}({\rm{\Psi }})+1}|.$$

Same as the Eq. (15), this inequality may have applications in other analysis of coherence by considering superposition effects.

The only difference between the first line and second line in Eq. (16) is also in the last term, which are −2|*αβ*| (*n* − 1) and −$2|\alpha \beta |\sqrt{({C}_{{l}_{1}}({\rm{\Phi }})+1)({C}_{{l}_{1}}({\rm{\Psi }})+1)}$ respectively. This comparison is dimension dependent as shown in Fig. 2. Note that since a measure of coherence is nonnegative, we compare the expressions in Eq. (16) and choose the maximum value as the lower bound.

### Robustness of coherence

As a quantifier of the advantage enabled by a quantum state in phase discrimination task, the robustness of coherence is defined and proved to be a full measure for the framework proposed in ref. 13. Robustness of coherence is shown to be an observable related to the notion of coherence witness^17, 18^. Given a fixed basis $\{{|i\rangle }_{i=1}^{n}\}$, where *n* is the dimension of Hilbert space. The definition of robustness of coherence of a state *ρ* is as following^17^:

18 $${C}_{R}(\rho )=\mathop{{\rm{\min }}}\limits_{\tau \in D({{\mathbb{C}}}^{d})}\,\{s\ge 0|\frac{\rho +s\tau }{1+s}=:\delta \in  {\mathcal I} \},$$

where $D({{\mathbb{C}}}^{d})$ is the convex set of density operators acting on a *n*-dimensional Hilbert space, and ${\mathcal I}$ is the set of all incoherent states. Notice that the robustness of coherence of a pure state |*ϕ*〉 satisfies^18^,

19 $${C}_{R}(\varphi )={C}_{{l}_{1}}(\varphi \mathrm{).}$$

From this equation, we can see the equivalence between robustness of coherence and *l*_1_ norm of coherence for pure states. The bounds are same as in Theorem 3 and Theorem 4.

### Coherence of superpositions for two states from orthogonal subspaces

Quantum states from orthogonal subspaces play an important role in quantum information and coding^36^. Here we consider the bounds for this special case. That is, the decomposition states (|Φ〉 and |Ψ〉) come from orthogonal subspaces.

**Corollary 1**. *Let* $|{\rm{\Phi }}\rangle ={\sum }_{i=1}^{n}\,{a}_{i}|i\rangle$, $|{\rm{\Psi }}\rangle ={\sum }_{i=1}^{n}\,{b}_{i}|i\rangle$ *be two states satisfying a*_*i*_ *b*_*i*_ = 0, $i=1,\ldots ,n$ (|Φ〉 *and* |Ψ〉 *belong to two orthogonal subspaces*), *and* $|{\rm{\Omega }}\rangle =\alpha |{\rm{\Phi }}\rangle +\beta |{\rm{\Psi }}\rangle$, ${|\alpha |}^{2}+{|\beta |}^{2}=1$. *Then the coherence of the superposition state* |Ω〉 *has the following bounds*:

(I) *for relative entropy of coherence*
  20 $${C}_{re}({\rm{\Omega }})={|\alpha |}^{2}{C}_{re}({\rm{\Phi }})+{|\beta |}^{2}{C}_{re}({\rm{\Psi }})+{h}_{2}({|\alpha |}^{2}),$$
  *where* |*α*|^2^ + |*β*|^2^ = 1 *and* $${h}_{2}(x)\equiv -x\,\mathrm{log}\,x-(1-x)\,\mathrm{log}\,(1-x)$$.
(II) *for l*_1_ *norm of coherence*

21 $$\begin{array}{c}{C}_{{l}_{1}}({\rm{\Omega }})\le \,{\rm{\min }}\{\begin{array}{l}{|\alpha |}^{2}{C}_{{l}_{1}}({\rm{\Phi }})+{|\beta |}^{2}{C}_{{l}_{1}}({\rm{\Psi }})+n|\alpha \beta |,\\ {|\alpha |}^{2}{C}_{{l}_{1}}({\rm{\Phi }})+{|\beta |}^{2}{C}_{{l}_{1}}({\rm{\Psi }})+2|\alpha \beta |\sqrt{({C}_{{l}_{1}}({\rm{\Phi }})+1)({C}_{{l}_{1}}({\rm{\Psi }})+1)},\end{array}\end{array}$$

*and*

22 $${C}_{{l}_{1}}({\rm{\Omega }})\ge {|\alpha |}^{2}{C}_{{l}_{1}}({\rm{\Phi }})+{|\beta |}^{2}{C}_{{l}_{1}}({\rm{\Psi }})+2|\alpha \beta |.$$

For the superposition of two states from two orthogonal subspaces, the relative entropy coherence is the sum of three terms: the average of the coherence of two states being superposed, the binary entropy of probability |*α*|^2^. Instead of bounds, this is an accurate expression as shown in Eq. (20). The maximum increase for coherence is bounded as following:

23 $${C}_{re}({\rm{\Omega }})-{|\alpha |}^{2}{C}_{re}({\rm{\Phi }})-{|\beta |}^{2}{C}_{re}({\rm{\Psi }})\le 1.$$

The bounds for *l*_1_ norm coherence of superposition considering orthogonal subspaces in Corollary 1 is tighter compared with the general case in Theorem 3 and 4. Specifically, the expression given in the first line of Eq. (21) is lowered and the expression given in Eq. (22) is raised.

## Discussion

Using the relative entropy of coherence, *l*_1_ norm of coherence and robustness of coherence, we give the upper and lower bounds for coherence of the superposition. Bounds for coherence of superpositions of multiple terms can be easily found by generalizing our methods.

Superposition is the root of both coherence and entanglement. Coherence is a property of an arbitrary quantum state, while entanglement is a property of a bipartite or multipartite state. In this sense, it is straightforward to understand that coherence is a more general quantum property than entanglement^20^. Entanglement of the superposition is closely linked to the entanglement of these two superposed states^28, 29, 31^. In this work, we have shown that the coherence of the superposition is intimately related to the coherence of these two superposed states. However, it is still unclear that the difference between entanglement of superpositions and coherence of superpositions. As shown in Tables 1 and 2, there exist strong similarities between coherence and entanglement for the analogy problem.

**Table 1:** Comparison between bounds on partial entropy of entanglement and relative entropy coherence for the superposition of two orthogonal states (Here $E({\varphi }^{AB})=S(T{r}_{A}|\varphi \rangle \langle \varphi |)$ is an entanglement measure of pure state *ϕ* ^*AB*^ ^28^).

**upper bound for partial entropy of entanglement** ^29^	**upper bound for relative entropy of coherence**
$\frac{1}{q}[pE({\rm{\Phi }})+\mathrm{(1}-p)E({\rm{\Psi }})+{h}_{2}(p)]$	$${\rm{\min }}\{\begin{array}{l}\frac{1}{q}f(p),\\ f(p)+\mathrm{2(1}-q)|\alpha ||\beta |\,\mathrm{log}(n-\mathrm{1)}+\mathrm{(1}-q)\end{array}$$
**lower bound for partial entropy of entanglement** ^29^	**lower bound for relative entropy of coherence**
$${\rm{\max }}\{\begin{array}{l}\frac{\mathrm{(1}-p){|\alpha |}^{2}}{1-p{|\alpha |}^{2}}E({\rm{\Phi }})-\frac{1-p}{p}E({\rm{\Psi }})-\frac{1}{p}{h}_{2}(p)\\ \frac{\mathrm{(1}-p){|\beta |}^{2}}{1-p{|\beta |}^{2}}E({\rm{\Phi }})-\frac{1-p}{p}E({\rm{\Psi }})-\frac{1}{p}{h}_{2}(p)\end{array}$$	$${\rm{\max }}\{\begin{array}{l}\frac{\mathrm{(1}-p){|\alpha |}^{2}}{1-p{|\alpha |}^{2}}{C}_{re}({\rm{\Phi }})-\frac{1-p}{p}{C}_{re}({\rm{\Psi }})-\frac{1}{p}{h}_{2}(p)\\ \frac{\mathrm{(1}-p){|\beta |}^{2}}{1-p{|\beta |}^{2}}{C}_{re}({\rm{\Phi }})-\frac{1-p}{p}{C}_{re}({\rm{\Psi }})-\frac{1}{p}{h}_{2}(p)\end{array}$$

**Table 2:** Comparison between bounds on concurrence and *l*_1_ norm coherence for the superposition of two orthogonal states (Here *E*_*C*_ is concurrence which is a measure of entanglement^31, 38^).

**upper bound for concurrence** ^31^	**upper bound for** ***l***_**1**_ **norm of coherence**
${|\alpha |}^{2}{E}_{C}({\rm{\Phi }})+{|\beta |}^{2}{E}_{C}({\rm{\Psi }})+2|\alpha \beta |$	$${\rm{\min }}\{\begin{array}{l}{|\alpha |}^{2}{C}_{{l}_{1}}({\rm{\Phi }})+{|\beta |}^{2}{C}_{{l}_{1}}({\rm{\Psi }})+\mathrm{2(}n-\mathrm{1)}|\alpha \beta |\\ {|\alpha |}^{2}{C}_{{l}_{1}}({\rm{\Phi }})+{|\beta |}^{2}{C}_{{l}_{1}}({\rm{\Psi }})+2|\alpha \beta |\sqrt{({C}_{{l}_{1}}({\rm{\Phi }})+1)({C}_{{l}_{1}}({\rm{\Psi }})+1)}\end{array}$$
**lower bound for concurrence** ^31^	**lower bound for** ***l***_**1**_ **norm of coherence**
${|\alpha |}^{2}{E}_{C}({\rm{\Phi }})-{|\beta |}^{2}{E}_{C}({\rm{\Psi }})-2|\alpha \beta |$ or ${|\beta |}^{2}{E}_{C}({\rm{\Psi }})-{|\alpha |}^{2}{E}_{C}({\rm{\Phi }})-2|\alpha \beta |$	$${\rm{\max }}\{\begin{array}{l}{|\alpha |}^{2}{C}_{{l}_{1}}({\rm{\Phi }})+{|\beta |}^{2}{C}_{{l}_{1}}({\rm{\Psi }})-\mathrm{2(}n-\mathrm{1)}|\alpha \beta |\\ {|\alpha |}^{2}{C}_{{l}_{1}}({\rm{\Phi }})+{|\beta |}^{2}{C}_{{l}_{1}}({\rm{\Psi }})-2|\alpha \beta |\sqrt{({C}_{{l}_{1}}({\rm{\Phi }})+1)({C}_{{l}_{1}}({\rm{\Psi }})+1)}\end{array}$$

From the expressions in Table 2, we can see that the upper bound and lower bound for coherence are symmetric about the statistical average coherence of the two superposed orthogonal states, which is defined as ${|\alpha |}^{2}{C}_{{l}_{1}}({\rm{\Phi }})+{|\beta |}^{2}{C}_{{l}_{1}}({\rm{\Psi }})$, and the green line as shown in Fig. 2(a). While the bounds for entanglement are asymmetric around the statistical average coherence of the two superposed orthogonal states defined as ${|\alpha |}^{2}{E}_{C}({\rm{\Phi }})+{|\beta |}^{2}{E}_{C}({\rm{\Psi }})$, and the green line as shown in Fig. 2(b). Here, we consider the case where our lower bound is valid, i.e. positive. In the following, we give an intuitive comparison between coherence and entanglement for Table 2 by taking a simple example. We focus on fluctuating ranges of *l*_1_ norm coherence and concurrence for the superposition of two orthogonal states. Here in order to make the discussion meaningful we consider the superposition of two bipartite orthogonal states.

*Example* 2: Let |Ψ〉 and |Φ〉 be two orthogonal states, defined by

24 $$|{\rm{\Psi }}\rangle =\frac{|00\rangle +|11\rangle }{\sqrt{2}}\,{\rm{and}}\,|{\rm{\Phi }}\rangle =\frac{|00\rangle +\sqrt{2}|01\rangle +|10\rangle -|11\rangle }{\sqrt{5}}.$$

**Figure 3:**
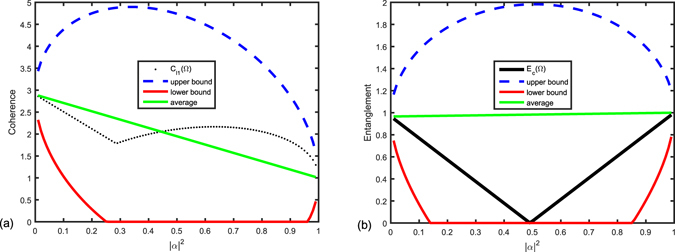
Bounds for *l*_1_ coherence (**a**) and concurrence (**b**) of the two states in Eq. (24). In sub-figure (**a**), the black dotted line is the actual values of *l*_1_ coherence, the blue dashed line is upper bound on *l*_1_ coherence, the red solid line is lower bound on *l*_1_ coherence, and the green line represents the statistical average coherence of the superposed states, ${|\alpha |}^{2}{C}_{{l}_{1}}({\rm{\Phi }})+{|\beta |}^{2}{C}_{{l}_{1}}({\rm{\Psi }})$. In sub-figure (**b**), the black dotted line is the actual values of concurrence, the blue dashed line is upper bound on concurrence, the red solid line is lower bound on concurrence, and the green line represents the statistical average entanglement of the superposed states, ${|\alpha |}^{2}{E}_{C}({\rm{\Phi }})+{|\beta |}^{2}{E}_{C}({\rm{\Psi }})$. Note that coherence and entanglement are both non-negative.

Their coherence and entanglement are depicted in Fig. 3. Evidently, the fluctuation of coherence is smoother than entanglement. The trend of change for coherence and entanglement with respect to the parameter |*α*|^2^ is not positive correlated with each other. Furthermore, there are two inflections points in the change of exact value of coherence while only one for entanglement considering the change of |*α*|^2^ in the case of example 2. Also in this example we can see that when entanglement of the superposition state disappears, the coherence still exists. From these observations, we can see that coherence is a kind of resource easier accessed compared with entanglement.

In some information processing tasks, such as coherence distillation, we are more concerned with the coherence of the state, rather than the state itself. When we only know the coherence value of |Φ〉 and |Ψ〉, and do not know what the specific state is, we can use our results to estimate the upper and lower bound of the superposition, then we can know that the least amount and the most amount of coherence we can distill from the superposition state.

Another interesting and challenging problem related to our work is the effects of superposition on the coherence of superposition of mixed states, which can be used to analyze the effects of de-coherence resulted from the interactions with environment.

## Methods

### Proof of the Theorem 1

We construct the state ${|\chi \rangle }^{SA}=\sqrt{p}{|{\rm{\Phi }}\rangle }^{S}{|0\rangle }^{A}+\sqrt{1-p}{|{\rm{\Psi }}\rangle }^{S}{|1\rangle }^{A}$, where we used an ancillary system A. The coherence of $|\chi \rangle$ can be expressed as $p{C}_{re}({\rm{\Phi }})+\mathrm{(1}-p){C}_{re}({\rm{\Psi }})+{h}_{2}(p)$. Through measuring ancillary system A with Kraus operators $\{|0\rangle (\cos \,\theta {e}^{i{\omega }_{1}}\langle 0|+\,\sin \,\theta {e}^{i{\omega }_{2}}\langle 1|),|1\rangle (-\,\sin \,\theta {e}^{-i{\omega }_{2}}\langle 0|+\,\cos \,\theta {e}^{-i{\omega }_{1}}\langle 1|)\}$, with probability $q={\Vert \sqrt{p}\cos \theta {e}^{i{\omega }_{1}}|{\rm{\Phi }}\rangle +\sqrt{1-p}\sin \theta {e}^{i{\omega }_{2}}|{\rm{\Psi }}\rangle )\Vert }^{2}$, the state becomes $|{\rm{\Omega }}\rangle |0\rangle =(\sqrt{\frac{p}{q}}\,\cos \,\theta {e}^{i{\omega }_{1}}|{\rm{\Phi }}\rangle +\sqrt{\frac{1-p}{q}}\,\sin \,\theta {e}^{i{\omega }_{2}}|{\rm{\Psi }}\rangle )|0\rangle$ and with probability 1 − *q*, the state becomes $|{\rm{\Gamma }}\rangle |1\rangle =(-\sqrt{\frac{p}{1-q}}\,\sin \,\theta {e}^{-i{\omega }_{2}}|{\rm{\Phi }}\rangle +\sqrt{\frac{1-p}{1-q}}\,\cos \,\theta {e}^{-i{\omega }_{1}}|{\rm{\Psi }}\rangle )|1\rangle$. This measurement is an incoherent operation and *C*_*re*_ is a coherence monotone^13^, we can get the following inequality

25 $$q{C}_{re}({\rm{\Omega }})+\mathrm{(1}-q){C}_{re}({\rm{\Gamma }})\le p{C}_{re}({\rm{\Phi }})+\mathrm{(1}-p){C}_{re}({\rm{\Psi }})+{h}_{2}(p\mathrm{).}$$

Since ${C}_{re}({\rm{\Gamma }})\ge 0$, we can get the following inequality

26 $${C}_{re}({\rm{\Omega }})\le \frac{1}{q}[p{C}_{re}({\rm{\Phi }})+\mathrm{(1}-p){C}_{re}({\rm{\Psi }})+{h}_{2}(p)]=\frac{1}{q}f(p\mathrm{).}$$

1. Now, we proof the  first line in Eq. (7) using the similar method in ref. 29 first. Here we set
  27 $$\frac{\alpha }{\Vert \alpha |{\rm{\Phi }}\rangle +\beta |{\rm{\Psi }}\rangle \Vert }=\sqrt{\frac{p}{q}}\,\cos \,\theta {e}^{i{\omega }_{1}}\,and\,\frac{\beta }{\Vert \alpha |{\rm{\Phi }}\rangle +\beta |{\rm{\Psi }}\rangle \Vert }=\sqrt{\frac{1-p}{q}}\,\sin \,\theta {e}^{i{\omega }_{2}}.$$
  It is straightforward to get
  28 $${|\alpha |}^{2}=\frac{p}{q}\,{\cos }^{2}\,\theta {\Vert \alpha |{\rm{\Phi }}\rangle +\beta |{\rm{\Psi }}\rangle \Vert }^{2}\,and\,{|\beta |}^{2}=\frac{1-p}{q}\,{\sin }^{2}\,\theta {\Vert \alpha |{\rm{\Phi }}\rangle +\beta |{\rm{\Psi }}\rangle \Vert }^{2}.$$
  Due to |*α*|^2^ + |*β*|^2^ = 1, and we can obtain
  29 $${\cos }^{2}\,\theta =\frac{\mathrm{(1}-p){|\alpha |}^{2}}{\mathrm{(1}-p){|\alpha |}^{2}+p{|\beta |}^{2}}$$
  Through substituting Eq. (29) into Eq. (28), and we can obtain
  30 $$q=\frac{p\mathrm{(1}-p)}{\mathrm{(1}-p){|\alpha |}^{2}+p{|\beta |}^{2}}{\Vert \alpha |{\rm{\Phi }}\rangle +\beta |{\rm{\Psi }}\rangle \Vert }^{2}.$$
  Finally, substitute Eq. (30) into Eq. (26), and the result can be proved
  31 $$\begin{array}{rcl}{C}_{re}({\rm{\Omega }}) & \le  & \frac{\mathrm{(1}-p){|\alpha |}^{2}+p{|\beta |}^{2}}{p\mathrm{(1}-p){\Vert \alpha |{\rm{\Phi }}\rangle +\beta |{\rm{\Psi }}\rangle \Vert }^{2}}[p{C}_{re}({\rm{\Phi }})+\mathrm{(1}-p){C}_{re}({\rm{\Psi }})+{h}_{2}(p)]\\  & = & \frac{1}{q}f(p),\end{array}$$
  with 0 < *p* < 1.□
2. The other bound in Eq. (7) is obtained through getting a tighter lower bound of ${C}_{re}({\rm{\Gamma }})$. Firstly, we introduce two lemmas on which the proof is based.

**Lemma 1**. (*Fannes*-*Audenaert inequality* ^37^) *Suppose ρ and σ are density matrices such that the trace distance is given by T*,

32 $$|S(\rho )-S(\sigma )|\le T\,\mathrm{log}(d-\mathrm{1)}+{h}_{2}(T),$$

*where d is the dimension of the Hilbert space*, *and* ${h}_{2}(x)=-x\,\mathrm{log}\,x-\mathrm{(1}-x)\,\mathrm{log}\,\mathrm{(1}-x)$.

**Lemma 2**. *Given two orthogonal states* |Φ〉, |Ψ〉, *and two complex number α*, *β satisfying* $\Vert \alpha |{\rm{\Phi }}\rangle +\beta |{\rm{\Psi }}\rangle \Vert =1$. *Let* $|{\rm{\Omega }}\rangle =\alpha |{\rm{\Phi }}\rangle +\beta |{\rm{\Psi }}\rangle$, $|{\rm{\Gamma }}\rangle =\alpha |{\rm{\Phi }}\rangle -\beta |{\rm{\Psi }}\rangle$, *and* ${|{\rm{\Omega }}\rangle }_{d}\langle {\rm{\Omega }}|$, ${|{\rm{\Gamma }}\rangle }_{d}\langle {\rm{\Gamma }}|$ *are the diagonal matrices of* $|{\rm{\Omega }}\rangle \langle {\rm{\Omega }}|$, $|{\rm{\Gamma }}\rangle \langle {\rm{\Gamma }}|$ *respectively*. *The trace distance between* ${|{\rm{\Omega }}\rangle }_{d}\langle {\rm{\Omega }}|$ *and* ${|{\rm{\Gamma }}\rangle }_{d}\langle {\rm{\Gamma }}|$ *satisfies*

33 $$T({|{\rm{\Omega }}\rangle }_{d}\langle {\rm{\Omega }}|,{|{\rm{\Gamma }}\rangle }_{d}\langle {\rm{\Gamma }}|)\le 2|\alpha \beta |.$$

Proof: Given $|{\rm{\Phi }}\rangle ={\sum }_{i=0}^{n-1}\,{a}_{i}|i\rangle$, and $|{\rm{\Psi }}\rangle ={\sum }_{i=0}^{n-1}\,{b}_{i}|i\rangle$,

34 $$\begin{array}{l}|{\rm{\Omega }}\rangle =\alpha |{\rm{\Phi }}\rangle +\beta |{\rm{\Psi }}\rangle =\sum _{i=0}^{n-1}\,(\alpha {a}_{i}+\beta {b}_{i})|i\rangle ,\\ |{\rm{\Gamma }}\rangle =\alpha |{\rm{\Phi }}\rangle -\beta |{\rm{\Psi }}\rangle =\sum _{i=0}^{n-1}\,(\alpha {a}_{i}-\beta {b}_{i})|i\rangle .\end{array}$$

Then we can get the diagonal matrices of |Ω〉〈Ω| and $|{\rm{\Gamma }}\rangle \langle {\rm{\Gamma }}|$ by deleting all off-diagonal elements as follow

35 $${|{\rm{\Omega }}\rangle }_{d}\langle {\rm{\Omega }}|=\sum \,(\alpha {a}_{i}+\beta {b}_{i})({\alpha }^{\ast }{a}_{i}^{\ast }+{\beta }^{\ast }{b}_{i}^{\ast })|i\rangle \langle i|,$$

36 $${|{\rm{\Gamma }}\rangle }_{d}\langle {\rm{\Gamma }}|=\sum \,(\alpha {a}_{i}-\beta {b}_{i})({\alpha }^{\ast }{a}_{i}^{\ast }-{\beta }^{\ast }{b}_{i}^{\ast })|i\rangle \langle i|\mathrm{.}$$

The trace distance between ${|{\rm{\Omega }}\rangle }_{d}\langle {\rm{\Omega }}|$ and ${|{\rm{\Gamma }}\rangle }_{d}\langle {\rm{\Gamma }}|$ is

37 $$T({|{\rm{\Omega }}\rangle }_{d}\langle {\rm{\Omega }}|,{|{\rm{\Gamma }}\rangle }_{d}\langle {\rm{\Gamma }}|)=\frac{1}{2}tr|R|.$$

Here, we denote $R={|{\rm{\Omega }}\rangle }_{d}\langle {\rm{\Omega }}|-{|{\rm{\Gamma }}\rangle }_{d}\langle {\rm{\Gamma }}|$ for short.

38 $$\begin{array}{rcl}|R| & = & \sqrt{{R}^{+}R}\\  & = & \sum \,\sqrt{\mathrm{4(}{\alpha }^{\ast }\beta {a}_{i}^{\ast }{b}_{i}+\alpha {\beta }^{\ast }{a}_{i}{b}_{i}^{\ast }{)}^{2}}|i\rangle \langle i|\\  & = & \sum \,\sqrt{16{|\alpha |}^{2}{|\beta |}^{2}{|{a}_{i}|}^{2}{|{b}_{i}|}^{2}}|i\rangle \langle i|\\  & = & \sum \,4|\alpha ||\beta ||{a}_{i}||{b}_{i}||i\rangle \langle i|.\end{array}$$

The upper bound of trace distance between ${|{\rm{\Omega }}\rangle }_{d}\langle {\rm{\Omega }}|$ and ${|{\rm{\Gamma }}\rangle }_{d}\langle {\rm{\Gamma }}|$ can be get as follows:

39 $$\begin{array}{rcl}T({|{\rm{\Omega }}\rangle }_{d}\langle {\rm{\Omega }}|,{|{\rm{\Gamma }}\rangle }_{d}\langle {\rm{\Gamma }}|) & = & \frac{1}{2}tr|R|\\  & = & 2|\alpha ||\beta |\sum \,|{a}_{i}||{b}_{i}|\\  & \le  & 2|\alpha ||\beta |\sum \,(\frac{{|{a}_{i}|}^{2}+{|{b}_{i}|}^{2}}{2})\\  & = & 2|\alpha ||\beta |,\end{array}$$

where the first inequality is due to fundamental inequality, and the last equality is due to the ${\sum }_{i}\,{|{a}_{i}|}^{2}={\sum }_{i}\,{|{b}_{i}|}^{2}=1$.□

We can get the following inequality from lemma 1 and 2

40 $$\begin{array}{ccc}|{C}_{re}({\rm{\Omega }})-{C}_{re}({\rm{\Gamma }})| & \le  & T({|{\rm{\Omega }}\rangle }_{d}\langle {\rm{\Omega }}|,{|{\rm{\Gamma }}\rangle }_{d}\langle {\rm{\Gamma }}|)\,{\rm{l}}{\rm{o}}{\rm{g}}(n-1)+{h}_{2}(T({|{\rm{\Omega }}\rangle }_{d}\langle {\rm{\Omega }}|,{|{\rm{\Gamma }}\rangle }_{d}\langle {\rm{\Gamma }}|))\\  & \le  & 2|\alpha ||\beta |\,{\rm{l}}{\rm{o}}{\rm{g}}(n-1)+1.\end{array}$$

Furthermore, we can get

41 $$\mathrm{(1}-q){C}_{re}({\rm{\Omega }})-\mathrm{(1}-q){C}_{re}({\rm{\Gamma }})\le \mathrm{2(1}-q)|\alpha ||\beta |\,\mathrm{log}(n-\mathrm{1)}+\mathrm{(1}-q),$$

and through shifting one term in LHS we can get

42 $$\mathrm{(1}-q){C}_{re}({\rm{\Gamma }})\ge \mathrm{(1}-q){C}_{re}({\rm{\Omega }})-\mathrm{2(1}-q)|\alpha ||\beta |\,\mathrm{log}(n-\mathrm{1)}-\mathrm{(1}-q).$$

Substitute Eq. (42) into Eq. (25), then we can get

43 $$\begin{array}{l}q{C}_{re}({\rm{\Omega }})+\mathrm{(1}-q){C}_{re}({\rm{\Omega }})-\mathrm{2(1}-q)|\alpha ||\beta |\,\mathrm{log}(n-\mathrm{1)}-\mathrm{(1}-q)\\ \begin{array}{rrl} & \le  & q{C}_{re}({\rm{\Omega }})+\mathrm{(1}-q){C}_{re}({\rm{\Gamma }})\le f(p\mathrm{).}\end{array}\end{array}$$

After simplification, we can get the result. Note that this upper bound is dimension dependent.□

### Proof of the Theorem 2

Let $|{\rm{\Omega }}\rangle =\alpha |{\rm{\Phi }}\rangle +\beta |{\rm{\Psi }}\rangle$. Through measuring the state ${|\chi ^{\prime} \rangle }^{SA}=\sqrt{p}{|{\rm{\Omega }}\rangle }^{S}{|0\rangle }^{A}+\sqrt{1-p}{|{\rm{\Psi }}\rangle }^{S}{|1\rangle }^{A}$, where we used an ancillary system A. The coherence of $|\chi ^{\prime} \rangle$ can be expressed as $p{C}_{re}({\rm{\Omega }})+\mathrm{(1}-p){C}_{re}({\rm{\Psi }})+{h}_{2}(p)$. We measure ancillary system A with Kraus operators $\{|0\rangle (\cos \,\theta {e}^{i{\omega }_{1}}\langle 0|+\,\sin \,\theta {e}^{i{\omega }_{2}}\langle 1|)$, $|1\rangle (-\,\sin \,\theta {e}^{-i{\omega }_{2}}\langle 0|+\,\cos \,\theta {e}^{-i{\omega }_{1}}\langle 1|)\}$, where $\beta =|\beta |{e}^{i({\omega }_{2}-{\omega }_{1})}$. With probability $t={\Vert \sqrt{p}\cos \theta {e}^{i{\omega }_{1}}|{\rm{\Omega }}\rangle +\sqrt{1-p}\sin \theta {e}^{i{\omega }_{2}}|{\rm{\Psi }}\rangle \Vert }^{2}$ the state becomes $|{{\rm{\Gamma }}}_{1}\rangle |0\rangle =(\sqrt{\frac{p}{t}}\,\cos \,\theta {e}^{i{\omega }_{1}}|{\rm{\Omega }}\rangle +\sqrt{\frac{1-p}{t}}\,\sin \,\theta {e}^{i{\omega }_{2}}|{\rm{\Psi }}\rangle )|0\rangle$ and with probability 1 − *t* the state becomes $|{{\rm{\Gamma }}}_{2}\rangle |1\rangle =(-\sqrt{\frac{p}{1-t}}\,\sin \,\theta {e}^{-i{\omega }_{2}}|{\rm{\Omega }}\rangle +\sqrt{\frac{1-p}{1-t}}\,\cos \,\theta {e}^{-i{\omega }_{1}}|{\rm{\Psi }}\rangle )|1\rangle$. This measurement is an incoherent operation and *C*_*re*_ is a coherence monotone^13^, we can get

44 $$t{C}_{re}({{\rm{\Gamma }}}_{1})+\mathrm{(1}-t){C}_{re}({{\rm{\Gamma }}}_{2})\le p{C}_{re}({\rm{\Omega }})+\mathrm{(1}-p){C}_{re}({\rm{\Psi }})+{h}_{2}(p\mathrm{).}$$

Since ${C}_{re}({{\rm{\Gamma }}}_{2})\ge 0$, we can obtain

45 $${C}_{re}({\rm{\Omega }})\ge \frac{1}{p}[t{C}_{re}({{\rm{\Gamma }}}_{1})-\mathrm{(1}-p){C}_{re}({\rm{\Psi }})-{h}_{2}(p)].$$

Through the following setting like in ref. 29, we can obtain $|{{\rm{\Gamma }}}_{1}\rangle =|{\rm{\Phi }}\rangle$

46 $$\alpha \sqrt{\frac{p}{t}}\,\cos \,\theta {e}^{i{\omega }_{1}}=1,$$

47 $$\beta \sqrt{\frac{p}{t}}\,\cos \,\theta {e}^{i{\omega }_{1}}+\sqrt{\frac{1-p}{t}}\,\sin \,\theta {e}^{i{\omega }_{2}}=0.$$

The parameters *α* and *β* satisfying Eqs (46) and (47) are as follows

48 $$\alpha =\sqrt{\frac{t}{p}}\frac{{e}^{-i{\omega }_{1}}}{\cos \,\theta },\,and\,\beta =-\sqrt{\frac{1-p}{p}}\frac{\sin \,\theta {e}^{i{\omega }_{2}}}{\cos \,\theta {e}^{i{\omega }_{1}}}\mathrm{.}$$

and

49 $${|\alpha |}^{2}=\frac{t}{p\,{\cos }^{2}\,\theta }\,and\,{|\beta |}^{2}=\frac{\mathrm{(1}-p)\,{\sin }^{2}\,\theta }{p\,{\cos }^{2}\,\theta }=\frac{{|\alpha |}^{2}\mathrm{(1}-p)\,{\sin }^{2}\,\theta }{t}.$$

We can obtain

50 $$t=\frac{p\mathrm{(1}-p){|\alpha |}^{2}}{1-p{|\alpha |}^{2}}\mathrm{.}$$

Substitute Eq. (50) into Eq. (45), then the results in Theorem 2 can be proved

51 $${C}_{re}({\rm{\Omega }})\ge \frac{\mathrm{(1}-p){|\alpha |}^{2}}{1-p{|\alpha |}^{2}}{C}_{re}({\rm{\Phi }})-\frac{1-p}{p}{C}_{re}({\rm{\Psi }})-\frac{1}{p}{h}_{2}(p),$$

with 0 < *p* < 1. As such, the another lower bound can be obtained by exchanging |Φ〉 and |Ψ〉.□

### Proof of the Theorem 3

Given two *n* dimensional states $|{\rm{\Phi }}\rangle ={\sum }_{i}^{n}\,{a}_{i}|i\rangle$ and $|{\rm{\Psi }}\rangle ={\sum }_{i}^{n}\,{b}_{i}|i\rangle$, where *a*_*i*_, *b*_*i*_ are complex numbers and satisfied $\sum \,{|{a}_{i}|}^{2}=\sum \,{|{b}_{i}|}^{2}=1$. Let $|{\rm{\Omega }}\rangle =\alpha |{\rm{\Phi }}\rangle +\beta |{\rm{\Psi }}\rangle$, and $\Vert {\rm{\Omega }}\Vert =1$.

1. Firstly, we prove the first upper bound in Eq. (14). From the definition of *l*_1_ norm of coherence Eq. (12). We have
  52 $$\begin{array}{rcl}{C}_{{l}_{1}}({\rm{\Phi }}) & = & \sum _{i\ne j}\,|{a}_{i}{a}_{j}|,\\ {C}_{{l}_{1}}({\rm{\Psi }}) & = & \sum _{i\ne j}\,|{b}_{i}{b}_{j}|.\end{array}$$
  and
  53 $$\begin{array}{rcl}{C}_{{l}_{1}}({\rm{\Omega }}) & = & \sum _{i\ne j}\,|(\alpha {a}_{i}+\beta {b}_{i})(\alpha {a}_{j}+\beta {b}_{j})|\\  & \le  & \sum _{i\ne j}\,({|\alpha |}^{2}|{a}_{i}{a}_{j}|+{|\beta |}^{2}|{b}_{i}{b}_{j}|+2|\alpha \beta ||{a}_{i}{b}_{j}|)\\  & = & {|\alpha |}^{2}\sum _{i\ne j}\,|{a}_{i}{a}_{j}|+{|\beta |}^{2}\sum _{i\ne j}\,|{b}_{i}{b}_{j}|+2|\alpha \beta |\sum _{i\ne j}\,|{a}_{i}{b}_{j}|\\  & = & {|\alpha |}^{2}{C}_{{l}_{1}}({\rm{\Phi }})+{|\beta |}^{2}{C}_{{l}_{1}}({\rm{\Psi }})+|\alpha \beta |\sum _{i\ne j}\,2|{a}_{i}{b}_{j}|,\end{array}$$
  where the first inequality is due to absolute value inequality. Successive application of the mean inequality, we will get
  54 $$2\sum _{i\ne j}\,|{a}_{i}{b}_{j}|\le (n-\mathrm{1)}(\sum _{i}\,{|{a}_{i}|}^{2}+\sum _{j}\,{|{b}_{j}|}^{2})\le \mathrm{2(}n-\mathrm{1),}$$
  which is the first line in Eq. (14).
2. Now, we prove the other upper bound in Eq. (14). From the definition of *l*_1_ norm of coherence Eq. (13), we have

55 $$\begin{array}{rcl}{C}_{{l}_{1}}({\rm{\Phi }}) & = & {(\sum _{i}|{a}_{i}|)}^{2}-1,\\ {C}_{{l}_{1}}({\rm{\Psi }}) & = & {(\sum _{i}|{b}_{i}|)}^{2}-1.\end{array}$$

Hence, we can obtain

56 $$\begin{array}{rcl}{C}_{{l}_{1}}({\rm{\Omega }}) & = & {(\sum _{i}|\alpha {a}_{i}+\beta {b}_{i}|)}^{2}-1\\  & \le  & {(\sum _{i}|\alpha {a}_{i}|+\sum _{i}|\beta {b}_{i}|)}^{2}-1\\  & = & {|\alpha |}^{2}{(\sum _{i}|{a}_{i}|)}^{2}+{|\beta |}^{2}{(\sum _{i}|{b}_{i}|)}^{2}+2|\alpha \beta ||(\sum _{i}\,|{a}_{i}|)(\sum _{i}\,|{b}_{i}|)|-1\\  & = & {|\alpha |}^{2}{C}_{{l}_{1}}({\rm{\Phi }})+{|\beta |}^{2}{C}_{{l}_{1}}({\rm{\Psi }})+2|\alpha \beta ||(\sum _{i}\,|{a}_{i}|)(\sum _{i}\,|{b}_{i}|)|.\end{array}$$

where the first inequality is due to absolute value inequality. Further, we have

57 $$|(\sum _{i}\,|{a}_{i}|)(\sum _{i}\,|{b}_{i}|)|=\sqrt{{(\sum _{i}|{a}_{i}|)}^{2}{(\sum _{i}|{b}_{i}|)}^{2}}=\sqrt{({C}_{{l}_{1}}({\rm{\Phi }})+1)({C}_{{l}_{1}}({\rm{\Psi }})+1)}.$$

The upper bound can be gained by substituting Eq. (57) into Eq. (56).□

### Proof of the Theorem 4

Given two *n* dimensional states $|{\rm{\Phi }}\rangle ={\sum }_{i=1}^{n}\,{a}_{i}|i\rangle$, and $|{\rm{\Psi }}\rangle ={\sum }_{i=1}^{n}\,{b}_{i}|i\rangle$, where *a*_*i*_, *b*_*i*_, are complex numbers and satisfy $\sum \,{|{a}_{i}|}^{2}=\sum \,{|{b}_{i}|}^{2}=1$. Let $|{\rm{\Omega }}\rangle =\alpha |{\rm{\Phi }}\rangle +\beta |{\rm{\Psi }}\rangle$, and $\Vert {\rm{\Omega }}\Vert =1$.

1. From the definition of *l*_1_ norm of coherence Eq. (12), we have
  58 $$\begin{array}{rcl}{C}_{{l}_{1}}({\rm{\Phi }}) & = & \sum _{i\ne j}\,|{a}_{i}{a}_{j}|,\\ {C}_{{l}_{1}}({\rm{\Psi }}) & = & \sum _{i\ne j}\,|{b}_{i}{b}_{j}|.\end{array}$$
  Thus, it has
  59 $$\begin{array}{rcl}{C}_{{l}_{1}}({\rm{\Omega }}) & = & \sum _{i\ne j}\,|\alpha {a}_{i}+\beta {b}_{i}||\alpha {a}_{j}+\beta {b}_{j}|\\  & \ge  & \sum _{i\ne j}\,(|\alpha {a}_{i}|-|\beta {b}_{i}|)(|\alpha {a}_{j}|-|\beta {b}_{j}|)\\  & = & \sum _{i\ne j}\,({|\alpha |}^{2}|{a}_{i}{a}_{j}|+{|\beta |}^{2}|{b}_{i}{b}_{j}|-2|\alpha \beta ||{a}_{i}{b}_{j}|)\\  & = & {|\alpha |}^{2}{C}_{{l}_{1}}({\rm{\Phi }})+{|\beta |}^{2}{C}_{{l}_{1}}({\rm{\Psi }})-|\alpha \beta |\sum _{i\ne j}\,2|{a}_{i}{b}_{j}|,\end{array}$$
  where the first inequality is due to absolute value inequality. Further, we find
  60 $$-2\sum _{i\ne j}\,|{a}_{i}{b}_{j}|\ge -(n-\mathrm{1)}(\sum _{i}\,{|{a}_{i}|}^{2}+\sum _{j}\,{|{b}_{j}|}^{2})\ge -\mathrm{2(}n-1).$$
  Therefore, the first lower bound in Theorem 4 can be obtained
2. From the definition of *l*_1_ norm of coherence Eq. (13), we have

61 $$\begin{array}{rcl}{C}_{{l}_{1}}({\rm{\Phi }}) & = & {(\sum _{i}|{a}_{i}|)}^{2}-1,\\ {C}_{{l}_{1}}({\rm{\Psi }}) & = & {(\sum _{i}|{b}_{i}|)}^{2}-1.\end{array}$$

Hence, we can obtain

62 $$\begin{array}{rcl}{C}_{{l}_{1}}({\rm{\Omega }}) & = & {(\sum _{i}|\alpha {a}_{i}+\beta {b}_{i}|)}^{2}-1\\  & \ge  & {(\sum _{i}|\alpha {a}_{i}|-\sum _{i}|\beta {b}_{i}|)}^{2}-1\\  & = & {|\alpha |}^{2}{(\sum _{i}|{a}_{i}|)}^{2}+{|\beta |}^{2}{(\sum _{i}|{b}_{i}|)}^{2}-2|\alpha \beta ||(\sum _{i}\,|{a}_{i}|)(\sum _{i}\,|{b}_{i}|)|-1\\  & = & {|\alpha |}^{2}{C}_{{l}_{1}}({\rm{\Phi }})+{|\beta |}^{2}{C}_{{l}_{1}}({\rm{\Psi }})-2|\alpha \beta |(\sum _{i}\,|{a}_{i}|)(\sum _{i}\,|{b}_{i}|).\end{array}$$

The second lower bound in Eq. (14) can be obtained by substituting the following Eq. into Eq. (62)

63 $$(\sum _{i}\,|{a}_{i}|)(\sum _{i}\,|{b}_{i}|)=\sqrt{{(\sum _{i}|{a}_{i}|)}^{2}{(\sum _{i}|{b}_{i}|)}^{2}}=\sqrt{({C}_{{l}_{1}}({\rm{\Phi }})+1)({C}_{{l}_{1}}({\rm{\Psi }})+1)}\mathrm{.}$$

### Proof of the Corollary 1

Given $|{\rm{\Phi }}\rangle ={\sum }_{i=1}^{n}\,{a}_{i}|i\rangle$, $|{\rm{\Psi }}\rangle ={\sum }_{i=1}^{n}\,{b}_{i}|i\rangle$ be two states satisfying *a*_*i*_ *b*_*i*_ = 0, and $|{\rm{\Omega }}\rangle =\alpha |{\rm{\Phi }}\rangle +\beta |{\rm{\Psi }}\rangle$, and ${|\alpha |}^{2}+{|\beta |}^{2}=1$.□

1. According to the conditions of Corollary 1, we can easily get
  64 $${|\alpha |}^{2}S({|{\rm{\Phi }}\rangle }_{d}\langle {\rm{\Phi }}|)+{|\beta |}^{2}S({|{\rm{\Psi }}\rangle }_{d}\langle {\rm{\Psi }}|)+h({|\alpha |}^{2})=S({|{\rm{\Omega }}\rangle }_{d}\langle {\rm{\Omega }}|).$$
  Thus, we can get Eq. (20) in terms of Eq. (6).
2. Let *m*_1_ and *m*_2_ are the number of nonzero probability amplitude of |Φ〉 and |Ψ〉 respectively, and *m*_1_ + *m*_2_ ≤ *n*. From the definition of *l*_1_ norm of coherence Eq. (12), we have

65 $$\begin{array}{rcl}{C}_{{l}_{1}}({\rm{\Omega }}) & = & \sum _{i\ne j}\,|(\alpha {a}_{i}+\beta {b}_{i})(\alpha {a}_{j}+\beta {b}_{j})|\\  & \le  & \sum _{i\ne j}\,({|\alpha |}^{2}|{a}_{i}{a}_{j}|+{|\beta |}^{2}|{b}_{i}{b}_{j}|+2|\alpha \beta ||{a}_{i}{b}_{j}|)\\  & = & {|\alpha |}^{2}\sum _{i\ne j}\,|{a}_{i}{a}_{j}|+{|\beta |}^{2}\sum _{i\ne j}\,|{b}_{i}{b}_{j}|+2|\alpha \beta |\sum _{i\ne j}\,|{a}_{i}{b}_{j}|\\  & = & {|\alpha |}^{2}{C}_{{l}_{1}}({\rm{\Phi }})+{|\beta |}^{2}{C}_{{l}_{1}}({\rm{\Psi }})+|\alpha \beta |\sum _{i\ne j}\,2|{a}_{i}{b}_{j}|,\end{array}$$

where the first inequality is due to absolute value inequality. Successive application of the mean inequality, we will get

66 $$2\sum _{i\ne j}\,|{a}_{i}{b}_{j}|\le {m}_{2}(\sum _{i}\,{|{a}_{i}|}^{2})+{m}_{1}(\sum _{j}\,{|{b}_{j}|}^{2})\le n,$$

We will get the first  upper bound by substituting Eq. (66) into Eq. (65).

Since the second upper bound Eq. (14) presented in Theorem 3 also works for this case, we can get the upper bound for the coherence of superposition of two states from two orthogonal subspaces as shown in Eq. (21).

In the following we give the lower bound for the coherence of superposition of two states from two orthogonal subspaces

67 $$\begin{array}{rcl}{C}_{{l}_{1}}({\rm{\Omega }}) & = & \sum _{i\ne j}\,|\alpha {a}_{i}+\beta {b}_{i}||\alpha {a}_{j}+\beta {b}_{j}|\\  & \ge  & \sum _{i\ne j}\,(|\alpha {a}_{i}|-|\beta {b}_{i}|)(|\alpha {a}_{j}|-|\beta {b}_{j}|)\\  & = & \sum _{i\ne j}\,({|\alpha |}^{2}|{a}_{i}{a}_{j}|+{|\beta |}^{2}|{b}_{i}{b}_{j}|-2|\alpha \beta ||{a}_{i}{b}_{j}|)\\  & = & {|\alpha |}^{2}{C}_{{l}_{1}}({\rm{\Phi }})+{|\beta |}^{2}{C}_{{l}_{1}}({\rm{\Psi }})-|\alpha \beta |\sum _{i\ne j}\,2|{a}_{i}{b}_{j}|.\end{array}$$

where the first inequality is due to absolute value inequality. Substitute Eq. (66) into Eq. (67), then we can get

68 $${C}_{{l}_{1}}({\rm{\Omega }})\ge {|\alpha |}^{2}{C}_{{l}_{1}}({\rm{\Phi }})+{|\beta |}^{2}{C}_{{l}_{1}}({\rm{\Psi }})-n|\alpha \beta |.$$

Another way to get the lower bound for the coherence of the superposition of two states from two orthogonal subspaces are as follows:

69 $$\begin{array}{rcl}{C}_{{l}_{1}}({\rm{\Omega }}) & = & \sum _{i,j}\,|(\alpha {a}_{i}+\beta {b}_{i})(\alpha {a}_{j}+\beta {b}_{j})-1|\\  & = & \sum _{i,j}\,({|\alpha |}^{2}|{a}_{i}{a}_{j}|+{|\beta |}^{2}|{b}_{i}{b}_{j}|+2|\alpha \beta ||{a}_{i}{b}_{j}|-1)\\  & = & {|\alpha |}^{2}{C}_{{l}_{1}}({\rm{\Phi }})+{|\beta |}^{2}{C}_{{l}_{1}}({\rm{\Psi }})+2|\alpha \beta |\sum \,\sum \,|{a}_{i}{b}_{j}|\\  & \ge  & {|\alpha |}^{2}{C}_{{l}_{1}}({\rm{\Phi }})+{|\beta |}^{2}{C}_{{l}_{1}}({\rm{\Psi }})+2|\alpha \beta |.\end{array}$$

Since the lower bound Eq. (16) presented in Theorem 4 also works for this case, we can get the lower bound for the coherence of superposition of two states from two orthogonal subspaces as follows:□

70 $$\begin{array}{ccc}{C}_{{l}_{1}}({\rm{\Omega }}) & \ge  & max\{\begin{array}{c}{|\alpha |}^{2}{C}_{{l}_{1}}({\rm{\Phi }})+{|\beta |}^{2}{C}_{{l}_{1}}({\rm{\Psi }})-n|\alpha \beta |,\\ {|\alpha |}^{2}{C}_{{l}_{1}}({\rm{\Phi }})+{|\beta |}^{2}{C}_{{l}_{1}}({\rm{\Psi }})-2|\alpha \beta |\sqrt{({C}_{{l}_{1}}({\rm{\Phi }})+1)({C}_{{l}_{1}}({\rm{\Psi }})+1)},\\ {|\alpha |}^{2}{C}_{{l}_{1}}({\rm{\Phi }})+{|\beta |}^{2}{C}_{{l}_{1}}({\rm{\Psi }})+2|\alpha \beta |,\end{array}\\  & = & {|\alpha |}^{2}{C}_{{l}_{1}}({\rm{\Phi }})+{|\beta |}^{2}{C}_{{l}_{1}}({\rm{\Psi }})+2|\alpha \beta |.\end{array}$$
